# Supplementary material for: One model to rule them all: Unification of voltage-gated potassium channel models via deep non-linear mixed effects modelling
Source: PLoS Comput Biol. 2026 Apr 27;22(4):e1013078. doi: 10.1371/journal.pcbi.1013078 (PMC13143182; doi:10.1371/journal.pcbi.1013078)
Supplement: S1 Text — (PDF) [file pcbi.1013078.s001.pdf]

S1 Text.

Hyper-parameter optimization

We have trained many different versions of the SciML HH model before settling on the one used in the main paper, including a Markov scheme with 3 states connected in different ways, a single neural network that returned multiple $\tau_i(V)$ and another one that returned $m_{i,\infty}(V)$ and many more. The model we settled on was performing the best on the validation data. Due to the cost of training a single model we used the following approach:

1. Split off a smaller data set, taking 2 cells from each $\mathrm{K_v}$ type
2. Set up a **unified** model $\mathcal{M}$ from a number of different combinations of hyper-parameters
   (a) number of gating particles $i \in [1, 2, 3]$
   (b) number of layers $\mathrm{N_L} \in [1, 2, 3]$
   (c) number of hidden units $\mathrm{N_H} \in [5, 10, 20]$
   (d) activation function act $\in [\tanh, \sigma, \mathrm{CeLU}]$
   (e) regularization constant $\lambda \in [10^{-2}, 10^{-3}, 10^{-4}, 10^{-5}]$
   (f) regularization type: $\mathrm{L_1}$ or $\mathrm{L_2}$
   (g) # of training epochs $[100, 200, 300, 600]$
3. Using conditional likelihood fit a model $\mathcal{M}$ and evaluate validation RMSE
4. Repeat setting up until options that make a significant different in training have been exhausted and select the best one

We opted to only do hyper-parameter optimization for the unified model reasoning that if an architecture would be powerful enough to fit a variety of different $\mathrm{K_v}$ types, it would also be powerful enough to fit individual $\mathrm{K_v}$ data since it is generally an easier task due to generally smaller variance within the data for individual channels. The final architecture we arrived (comparison of different architectures not shown) at was $i = 2$, $\mathrm{N_L}= 2$, $\mathrm{N_H}= 5$ with tanh nonlinearity using $\mathrm{L_1}$ regularization with $\lambda = 10^{-4}$ and 300 training epochs.

Having settled on an architecture, we trained all the models using it. However, for some $\mathrm{K_v}$ types we had to use multi-start optimization, using multiple different seeds for the initial parameters and restarting optimization, because the individual $\mathrm{K_v}$ models suffered from bad local optima. The number of different seeds used ranged from 1 to 15. Having obtained models that trained properly, we investigated their results further by saving model parameters every 15 iterations and evaluating the validation loss at those points, selecting the set of parameters that showed the lowest validation error, essentially doing early stopping (see Fig 1).

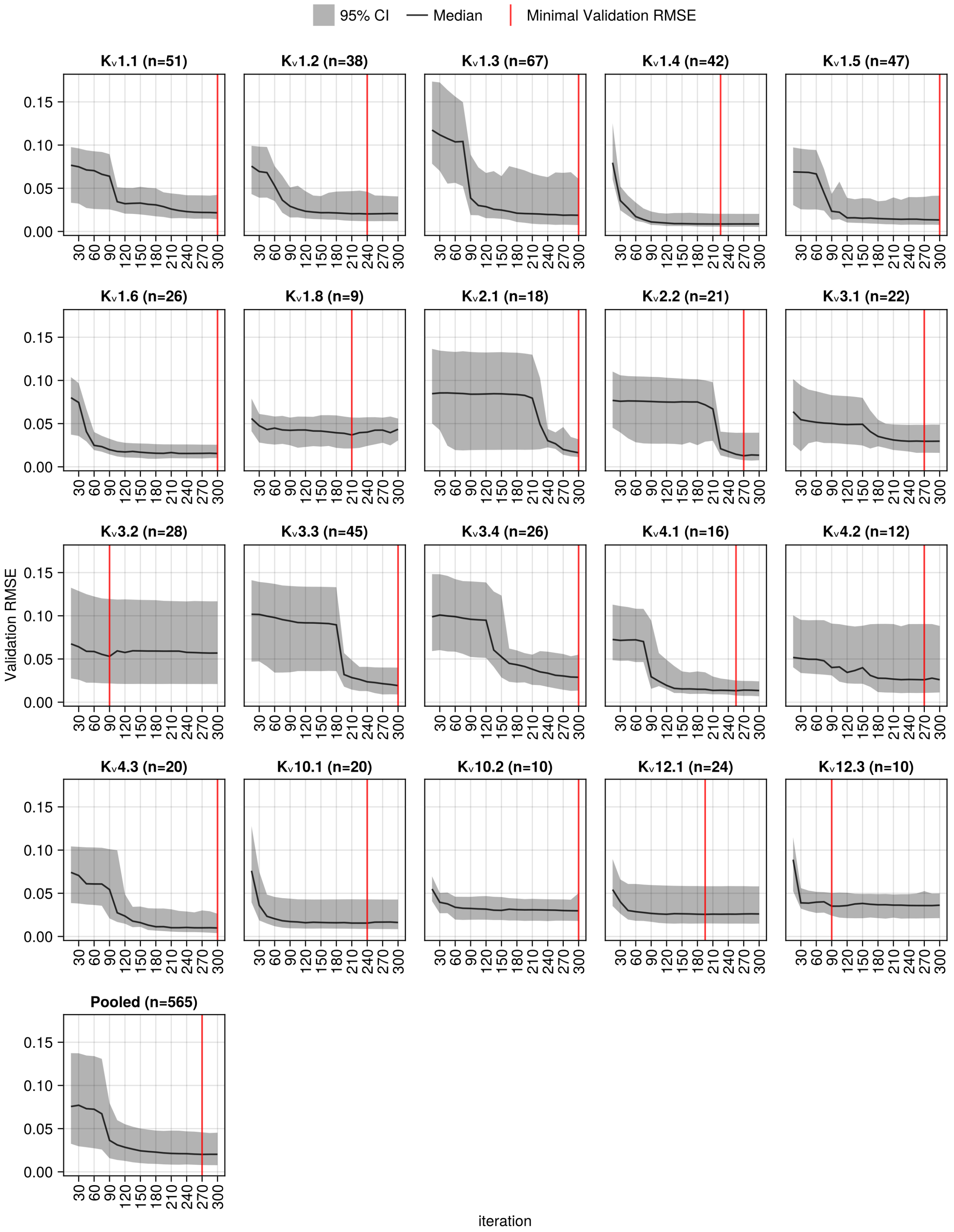

Fig 1. Early stopping evaluation of different individual and the unified K_v model. The red line denotes the iteration at which the validation RMSE was minimal, the black line is the median validation RMSE and the shaded area is the 95% confidence interval for the validation RMSE.
