# Supplementary material for: One model to rule them all: Unification of voltage-gated potassium channel models via deep non-linear mixed effects modelling
Source: PLoS Comput Biol. 2026 Apr 27;22(4):e1013078. doi: 10.1371/journal.pcbi.1013078 (PMC13143182; doi:10.1371/journal.pcbi.1013078)
Supplement: S2 Text — (PDF) [file pcbi.1013078.s002.pdf]

S2 Text.

Additional information about the baseline K_v models used.

Model constants equations for $K_v1.1$ from Ranjan et al. [1]

$$
\begin{aligned}
temp &= T + \eta \\
h_{\infty Q10} &= 0.032 \cdot temp - 0.365 \\
h_{\tau Q10} &= 2.7^{\frac{temp-25.0}{10.0}} \\
vBreak &= -46.7 \\
amp1 &= 52.7 \\
amp2 &= 15.98 \\
vh1 &= -49.87 \\
vh2 &= -41.64 \\
slope1 &= 5.0 \\
slope2 &= 24.99 \\
offset &= 0.9 \\
E_{ion} &= \frac{8.314 \cdot (T + 273.15)}{96490.0} \cdot \log\left(\frac{4}{151}\right) \cdot 1000.0 \\
\tilde{V} &= 80 - E_{ion} \\
h_{\infty} &= 1 - h_{\infty Q10} + \frac{h_{\infty Q10}}{1 + e^{\frac{V+31.0}{5.256}}} \\
h_{\tau} &= \frac{86.86 + \frac{408.78}{1+e^{\frac{V+13.6}{7.46}}}}{h_{\tau Q10}} \\
m_{\infty} &= \frac{1}{1 + e^{\frac{V+14.16}{-10.15}}} \\
m_{\tau Q10} &= \left(7.54 \cdot e^{\frac{-V}{379.7}} \cdot e^{\frac{-temp}{35.66}}\right)^{\frac{temp-25.0}{10.0}} \\
sigswitch &= \frac{1.0}{1 + e^{\frac{V-vBreak}{3.0}}} \\
sig1 &= \frac{sigswitch \cdot amp1}{1 + e^{\frac{V-vh1}{-slope1}}} \\
sig2 &= (1 - sigswitch) \cdot offset + \frac{amp2 - offset}{1 + e^{\frac{V-vh2}{slope2}}} \\
mTauFunc &= sig1 + sig2 \\
m_{\tau} &= \frac{mTauFunc}{m_{\tau Q10}} \\
\frac{\mathrm{d} \cdot V(t)}{\mathrm{d}t} &= 0.0 \\
\frac{\mathrm{d} \cdot m(t)}{\mathrm{d}t} &= \frac{m_{\infty} - m}{m_{\tau}} \\
\frac{\mathrm{d} \cdot h(t)}{\mathrm{d}t} &= \frac{h_{\infty} - h}{h_{\tau}}
\end{aligned} \tag{1}
$$

Table 1. Comparison of performance between different models on the test data set.

Channel type	Source publication	Link to model definition	Data the model is tuned to
$K_v1.1$	Ranjan et al. [1]	Equation (1)	https://channelpedia.epfl.ch/ionchannels/1#expdata
$K_v1.2$	Shen et al. [2]	https://modeldb.science/97860?tab=2&file=rejuvenation/mod/kv1_gp.mod	Data in publication
$K_v1.5$	Masoli et al. [3]	https://modeldb.science/229585?tab=2&file=purkinjecell_2015/mod_files/Kv15.mod	Feng et al. [4]
$K_v3.1$	Wang et al. [5]	https://modeldb.science/3454?tab=2&file=kv31model/ht.mod	Data in publication
$K_v3.3$	Beining et al. [6]	https://modeldb.science/231818?tab=2&file=BeiningEtAl2017nrn/lib_mech/Kv33.mod	Tuned in the publication
$K_v3.4$	Beining et al. [6]	https://modeldb.science/231818?tab=2&file=BeiningEtAl2017nrn/lib_mech/Kv34.mod	Tuned in the publication
$K_v4.3$	Akemann and Knopfel et al. [7]	https://modeldb.science/80769?tab=2&file=AkemannKnopfelPurkinje_cell_model/Kv4.mod	Sacco et al. [8]

References

1. Ranjan R, Logette E, Marani M, Herzog M, Tâche V, Scantamburlo E, et al. A kinetic map of the homomeric voltage-gated potassium channel (Kv) family. Frontiers in Cellular Neuroscience. 2019;13. doi:10.3389/fncel.2019.00358.

2. Shen W, Hernandez-Lopez S, Tkatch T, Held JE, Surmeier DJ. Kv1.2-Containing K+ channels regulate subthreshold excitability of striatal medium spiny neurons. Journal of Neurophysiology. 2004;91(3):1337–1349. doi:10.1152/jn.00414.2003.

3. Masoli S, Solinas S, D'Angelo E. Action potential processing in a detailed Purkinje cell model reveals a critical role for axonal compartmentalization. Frontiers in Cellular Neuroscience. 2015;9. doi:10.3389/fncel.2015.00047.

4. Feng J, Xu D, Wang Z, Nattel S. Ultrarapid Delayed Rectifier Current Inactivation in Human Atrial Myocytes: Properties and Consequences. American Journal of Physiology-Heart and Circulatory Physiology. 1998;275(5):H1717–H1725. doi:10.1152/ajpheart.1998.275.5.H1717.

5. Wang LY, Gan L, Forsythe ID, Kaczmarek LK. Contribution of the Kv3.1 potassium channel to high-frequency firing in mouse auditory neurones. The Journal of Physiology. 1998;509(1):183–194. doi:10.1111/j.1469-7793.1998.183bo.x.

6. Beining M, Mongiat LA, Schwarzacher SW, Cuntz H, Jedlicka P. T2N as a new tool for robust electrophysiological modeling demonstrated for mature and adult-born dentate granule cells. eLife. 2017;6:e26517. doi:10.7554/eLife.26517.

7. Akemann W, Knöpfel T. Interaction of Kv3 potassium channels and resurgent sodium current influences the rate of spontaneous firing of Purkinje Neurons. The Journal of Neuroscience. 2006;26(17):4602–4612. doi:10.1523/JNEUROSCI.5204-05.2006.

8. Sacco T, Tempia F. A-Type Potassium Currents Active at Subthreshold Potentials in Mouse Cerebellar Purkinje Cells. The Journal of Physiology. 2002;543(2):505–520. doi:10.1113/jphysiol.2002.022525.
