# Supplementary material for: One model to rule them all: Unification of voltage-gated potassium channel models via deep non-linear mixed effects modelling
Source: PLoS Comput Biol. 2026 Apr 27;22(4):e1013078. doi: 10.1371/journal.pcbi.1013078 (PMC13143182; doi:10.1371/journal.pcbi.1013078)
Supplement: Attachment — Submitted filename: rebuttal_letter.pdf [file pcbi.1013078.s004.pdf]

School of informatics

THE UNIVERSITY OF EDINBURGH

6 December 2025

INSTITUTE *for* ADAPTIVE *and* NEURAL COMPUTATION

SCHOOL *of* INFORMATICS
The University of Edinburgh
The Informatics Forum
10 Crichton Street
Edinburgh EH8 9AB

Telephone 0131 650 3099
Fax 0131 650 6899
E-mail D.Linkevicius@sms.ed.ac.uk

Quentin Clairon, PhD
Guest Editor
PLOS Computational Biology

Hugues Berry
Section Editor
PLOS Computational Biology

Manuscript submission: "One model to rule them all: unification of voltage-gated potassium channel models via deep non-linear mixed effects modelling"
Domas Linkevicius, Angus Chadwick, Melanie I. Stefan & David C. Sterratt

Dear Dr Clairon and Prof Berry,

We are grateful to the Reviewers for their insightful comments and observations regarding our manuscript. Unfortunately, in-between the submission of the paper and the reception of the reviews we have lost the specific data we used to train and evaluate the models. In order to address some of the comments made by the reviewers we had to re-download the data from Channelpedia, and the re-downloaded data set in some cases slightly deviated from the original data, potentially either due to a few additional cells added for certain channel families or incomplete downloading in the original data set. The discrepancies are slight (on the order of 10s of additional sweeps to a data set of thousands of sweeps) and would not have impacted the model training in a significant manner. Therefore, in the file showing the differences between the original and the revised manuscripts the table showing the statistical test values, which, in principle, should not have any differences in the comparisons between the unified and individual SciML models, contains some numerical differences due to a combination of the models trained on the original data set being evaluated to an almost identical re-downloaded data, evaluated on a different machine and platform. To guard against such occurrences in the future and to promote reproducibility we have uploaded the re-downloaded data to a storage platform, as well as listed the cell IDs with links to Channelpedia in a supplemental file. We will now provide a point-by-point response to the comments provided the reviewers.

Reviewer #1 wrote:

"0. After the Introduction and up to the Methods section, the authors only mention what they do, but they don't explain why or from what references they draw their writing. This is particularly true of the parameters chosen, the methods used, and the general sequence of the process and the document. Some explanations are vague. It would be ok if some additional figures could be included to be clearer to the readers."

- We have now included additional references to previous work similar to ours, particularly past publications that used mixed-effects frameworks and machine learning.

"1. Please indicate the main characteristics of the data, to prevent the reader from obtaining

the reference." "2. Minimum, maximum, average of cell recordings?"

- We have added an additional table (numbered Table 1) with descriptive statistics of the data.

"3. WHAT is the 'sufficient' number of recordings ('sufficient' with respect to what and why?)"

- We have amended the wording in the text to be more precise.

"4. From (3): Why at those specific temperatures?"

- We clarified that those temperatures were the only ones available in the Channelpedia data repository.

"5. Authors provide and describe parameters with no rationale nor references. WHY do you work exactly with those values?"

- We are not clear which parameters the reviewer is referring to, but we would be happy to address this point if more details are specified.

"6. Part: Data Processing. Authors claim that: 'In order to make the data in Ranjan et al. [4] suitable for model training we undertook 166 a series of data processing steps (Figure 1). First, we filtered out inconsistent data. We 167 then applied a time series smoothing algorithm to reduce the noise levels, followed by 168 setting the current baseline to 0, normalization of the current, rescaling, exclusion of 169 systematic data artifacts and downsampling'.

Again, why those numbers and WHAT IS THE SMOOTHING algorithm? Why those parameters?"

- We clarified that more details about each data processing step are given in the sections that describe them and that the parameters were chosen such that the data would be visually as similar to the originally used data, except for immediately identifiable artefacts.

"7. WHY this...? All data processing, modelling and 170 analyses in this paper were performed using the Julia v1.10.4 programming 171 language."

- We added additional justification why Julia was used – speed of ODE solvers, presence of software packages that integrate NLME and ML functionalities.

"8. Why to use MAPE and not other measure or more than 1 measure? Why 1 measure is ok and why only one parameter is ok? How can you justify this?"

- We added additional reasoning why MAPE was used, providing references showing that it is an established metric in forecasting. Using more than one metric was deemed to not be necessary and potentially resulting in conflicts when the metrics are contradictory.

"9. Authors say that: "The dynamical parameters pn are then fed into the structural model (e.g. an ordinary 301 differential equation (ODE) system)". But where is the ODE system?"

- The ODE system is given in Equation (3) just below the referenced text, we clarified that u' denotes the time derivative.

"10. From (9), why not to use PDEs instead of ODEs? A good discretization could yield good results."

- Usage of PDEs in this context, especially in compartmental modelling of neurons, is outside of the scope of the present work. In the current work the cells conducting current have regular shapes and therefore can be approximated as points, so usage of PDEs is not necessary.

"11. From (9,10), how do you know that the set of ODE actually have a solution?"

- The reviewer correctly notes that the ODEs do not have an analytical solution, but that is not a significant problem as usage of numerical solvers in such modelling applications is standard practice.

"12. Depending of the parameters of the ODE, the ODE may reflect some ill-posed issue, particularly with small values of the coefficients."

- It is true that the resulting ODE system may be stiff, but this is addressed by using an autoswitching ODE solver that uses a stiff solver when the parameters result in a stiff system, and a non-stiff solver when the parameters produce a non-stiff system.

"13. Line 308: What do you mean with: 'the appropriate variables'?"

- We clarified the phrasing in this part of the text.

"14. Eq. 4 implies a lot of things but you do not explain anything else."

- We would be happy to address any implications stemming from this equation not mentioned in the text, if the reviewer could be a bit more specific about what is missing.

"15. How do ensure that your data has a joint Gaussian function?"

- We do not require that the data has a joint Gaussian function, as the Gaussian observational model is per-point, rather than joint, as indicated by the subscript n in Eq. 4. Also, Figure 8 provides favourable diagnostics that the error model we use is appropriate.

"16. About eq. (4), it has been proven that the uncertainty directly from Gaussian Processes Regression is irrelevant to the cohort heterogeneity in general. What happens in your case about this?"

- The only source we can find for this finding is in Xu et al (2021, "The pitfalls of using Gaussian Process Regression for normative modeling", PLoS ONE) who state "we show that the uncertainty directly from Gaussian Processes Regression is irrelevant to the cohort heterogeneity in general." However, the "cohort heterogeneity" modelled in that paper is different from the problem manifest in the data we are analysing. It is not clear to us how Gaussian Process Regression relates to to universal differential equations in an NLME context, as considered in our work.

"17. In real life, returns are not symmetrically distributed and exhibit extremes not captured well by the bell-shaped curve of the Gaussian model."

- We are not sure what the "returns" referred to here are, though this statement does appear to apply to financial scenarios, see, e.g., this article about the use of the Gaussian in finance, which states " In real life, returns are not symmetrically distributed and exhibit extremes not captured well by the bell-shaped curve of the Gaussian model." (`https://campus.datacamp.com/courses/financial-analytics-in-google-sheets/`

`monitoring-the-distribution-of-returns?ex=14`). **That said, it is true that in NLME applications a proportional, rather than additive error model is used, accounting better for extreme values. However, it is necessary only when the extreme values are orders of magnitude apart, which was not the case in the current data set.**

"18. Line 314: Maximum likelihood estimates can be heavily biased for small samples, thus the optimality properties may not apply for small samples. Maximum likelihood can be sensitive to the choice of starting value. What happens with yours?"

- We believe that the small sample regime does not apply to our case, since we have a large amount of data compared to general NLME applications. Also, our work is concerned with applying the NLME methodology to model the Kv gating, for the analysis usage of ML estimates under various data regimes we would refer the reader to the source publication for `Pumas.jl`.

"19. The optimization problem should formally be posed."

- The optimization problem is formally posed in equations 5 through 7 (as it is multi-stage optimization).

"20. How do you guarantee, in advance, that such optimum value will be found? (global, local...)."

- Guaranteeing that in advance for neural network based modelling is generally not possible, but requires exploration of hyper-parameters, which we show in S1 appendix. We can say with confidence that any minima are local, rather than global.

"21. How to be sure that the NN algorithm actually converges?"

- As shown in S1 appendix, since the training loss does not decrease with further training iterations, this means that it has converged locally.

"22. What is the computational and algorithmic complexity of the whole algorithm?"

- In this work we apply the NLME methodology, for features of the algorithm, such as its complexity, we refer the reader to the `Pumas.jl` source publication.

"23. Why not to conduct a statistical test to test the classification results?"

- Could the reviewer be more specific on which results warrant statistical tests pertinent to classification, as we do not believe we have conducted any classification in this work.

"24. From (16-18): Unlike ML estimators, the MAP estimate is not invariant under reparametrization. Switching from one parametrization to another involves introducing a Jacobian that impacts on the location of the maximum. In contrast, Bayesian posterior expectations are invariant under reparametrization. Please comment about this."

- In this work we do not endeavour to estimate parameters, as neural network parameters are not inherently physically meaningful. Therefore, while the reviewer raises a valid and interesting point, it is not immediately relevant to the current work.

Reviewer #2 wrote:

"The authors provide a good introduction into the usage of HH models the challenges and the state of the art. However, one of the key contributions, namely the modelling using neural networks and its application in combination with first prior models is not mentioned at all. Even though not many papers investigate these model types in combination with NLME models, its application in general scientific machine learning has been treated in various field. I advise that the authors give a brief overview on these developments to allow the reader to gain an overview on this topic."

- We have added an additional paragraph in the introduction, discussing various other applications that combine mixed effects modelling and machine learning generally, as well as NLME and SciML in particular.

"p. 4, 138 is AP properly introduced somewhere? If not, even if not used, I think it would be useful to get a further explanation on why this protocol can be dropped. Is this due to the hope the neural network is able to account for this? Is this common practice?"

- AP and Ramp protocols are not dropped due to theoretical considerations or benefits, but practical limitations – it is not possible to implement them in the software used. Since `Pumas.jl` is primarily oriented towards pharmacokinetics and pharmacodynamics modelling, it does not allow negative time-varying stimulation, which makes the implementation of the action potential and ramp protocols currently impossible. Comment added to this effect.

"p.5, 165 If the code for the data preprocessing available in the linked repository, consider mention the repo here."

- Added an extra mention of the repository.

"p. 11, 368 Why did the authors decide to use the same architecture for the individual and unified SciML model?"

- The primary reason for using the same architectures is parsimony, wanting to have the minimal model that would fit all the data. It is undoubtedly possible to have a larger model for the unified SciML model, but that introduces the risk of different parts of the larger model fitting different channel types, rather than extracting a more universal set of representations that a smaller model is more likely to do. Comment added to this effect.

"p. 11, 352 In general the authors go through great length of introducing methodology used. However, I find that the information on inputs and outputs of the neural network(s) is a little hard to find for such a prominent part of the paper. Consider to use proper equations instead of inlining here."

- Changed to proper equations instead of inlining.

"p. 14, Fig. 3 I am honestly surprised to see the performance of the classical model structure over the data. Even without a direct comparison I would not use this model. Are there more elaborate, first principle model better explaining the data?"

We revisited the simulations that we did and found that there was a mistake in setting the initial conditions of the classical HH models, setting the gating variables to zero, rather than to $\mathbf{m_\infty(V_{rest})}$. We have corrected for this error and the classical

model behaviour now looks more reasonable. As an additional check we reproduced Figure 8A from Ranjan et al. (Fig 3 A-C) to show that our implementation of their model is correct. We recalculated the statistics and this error did not qualitatively change the results. Fig 3 A-C shows that the model can fit cells close to the median kinetics, but we show that when we look at across cells the model can perform poorly either due to not having random effects and potentially due to suboptimal structure.

"p. 19, 501 ff. The conclusion drawn for the individual and unified model are correct. However, due to the training the expected benefit might vanish, given that the individual models are not able to specialize."

- We don't follow this comment, as individual models are by definition specialised, since they are trained on the data from a single type of a Kv channel and training to fit that data is what makes them specialised to that channel type. As we're not clear how we could improve the manuscript, we've not made any changes.

"p. 22, Fig. 8 I really like the style of these goodness of fit visualizations, which where new to me with the contour plots. However, I find it challenging to visualize the implication quickly due to the choice of a the colour scale. The authors might choose to switch to a colourized colour scale for the derivation and indicate the LOESS, OLS and identity relation (which overlay anyway) using black, red, or white?"

- We are glad the reviewer likes the plots, which took some experimentation to get to the current state. However, after further consideration, we believe that the currently used greyscale is more accessible and simpler to parse than a colourized scale, which would necessitate choosing of a colour vision deficient-friendly palette, which would not leave many good options for the other three qualitatively different lines.

"- p. 27 (There are multiple ways...) While the additional comparison between the different software packages is certainly informative, I propose that the authors move this comparison a) into section METHODS and maybe add a tabular summary or b) they move it to the back of the discussion given that it seems to divide two parts concerning the investigated models."

- As suggested, shifted the paragraph in question to be later in the text to improve the flow of text.

Reviewer #3 wrote:

"Line 335-343 Are these preliminary tests shown somewhere? How is this initial quality of fitting to the data measured? If the models are described to be a reasonably good baseline, it should be justified how. It would also be nice/useful if the authors could include the HH models (at least in the supplement) for readers to compare them with the provided NLME models generated by the authors."

- We provided a clarification of what the preliminary test was. We also added an extra appendix with links to the ModelDB files which were used as the source from which the models were ported to Julia and `Pumas.jl`, as well as data sources to which the models are said to be fitted.

"(Figure 3 and Figure 4). In tandem with the previous comment, why is this particular classical HH model chosen/utilized here? Can these HH model parameters be refitted to the data provided

here (keeping the identical structure but refitting the rate constants)? Or at least refit to an average of the data? The HH models seem to perform so poorly that their initial structure itself (i.e. the number of gates or types of gates) is incorrect based on the data provided. That is to say, if a model without inactivation or deactivation kinetics built into the HH/Markov scheme was used to fit data that clearly exhibits this property, then the original model should not be considered an accurate/useful/good model. This indicates that comparing the SciML model to this particular HH model yields an unfair comparison of model types as clearly the SCiML model with an inactivation component will do better on data that inactivates than an HH model that is incapable of inactivating. What data were the HH models initially tested on such that they were deemed to be reasonable models (presumably at some point they fit some data well)? What temperature is this data at and at what temperature is the original data the model was fitted to? It seems that Kv1.1 channels exhibit inactivation at higher temperatures but not for lower temperatures. Is it possible that the original HH model is meant to be used for a lower temperature range (where there isn't inactivation) but not at higher temperatures? In the very least, these comparisons should be done at other temperatures (or if there are multiple temperatures within this plot that should be made clear). Also more than just the response to a +80 voltage step should be shown. +80 reaches a voltage value that is non-physiologically relevant and thus might further exacerbate the poor performance of the unfit HH model. The summary plot in Figure 4 uses −10, and it is unclear why this wasn't the value chosen for figure 3. Were comparisons between models conducted across all Voltage ranges?"

-In Figure 3 the $K_{v1.1}$ model from Ranjan et al. model is utilized because it is the only model that is tuned to nominally the same data source, therefore it is the fairest comparison possible. In Figure 4 the given set of models is used because these are the best models we could find in a non-exhaustive ModelDB search over models which were nominally for single channel currents, rather than currents that are combinations from multiple channels. Most of these models match the unified SciML model structure in broad terms: all models have both activation and inactivation, except for $K_{v1.5}$ which has two inactivation particles, along with an activating one. All models included adjustments of functions due to temperature, specifically, the $K_{v1.1}$ model from Ranjan et al. model takes into account the temperature dependencies. We have incorporated the points made here in the Results and Methods section.

Whether the chosen classical HH model parameters can be refitted to the Channelpedia data is an interesting question. Significant effort would need to be put into achieving this, with uncertain outcomes in cases where some parts of the classical models may be misspecified (e. g. temperature dependence modelling or functional forms used). Moreover, we can not claim that there is no HH model with random effects may fit the data as well as our SciML models, but developing such models would be a significant amount of work because the current most performant NLME/SciML software (`Pumas.jl` and `DeepPumas.jl`) is not well-integrated with optimization techniques that would be required to search the combinatorial space of gate configurations and functional forms used to model their rates of change, necessitating manual exhaustive search. Moreover, the whole point of the paper is to circumvent the classical HH modelling and the larger number of decisions that need to be made to achieve a good fit to the data, replacing that with a more flexible data-driven methodology.

We agree with the point that if a model without inactivation or deactivation kinetics

built into the HH/Markov scheme was not used when data clearly exhibits this, then the model should not be considered accurate (even though we would argue that this generally was not the case with the models we used). We expanded on the structure of the baseline models used in the methods. We also agree that making conclusions about classical HH models on kinetically heterogeneous data is an unfair comparison and that is why we add random effects to these models.

Figure 4 does not use only Activation at -10mV; this voltage is given as an example of what a sweep is, and the figure all the processed data, i. e. all protocols and all sweeps. We have added a clarification to the caption.

"Lines 35-36 Citations or more explanation would be helpful for the statements "exhibit extreme behaviors" and "numerically unstable". Which functional forms are being discussed here from which papers? Is this in reference to the HH equations themselves, or just other models which have been fit using an $m^x h^y$ paradigm. Are the equations utilized and referenced here as "unstable" properly derived from statistical physics as boltzmann equations consisting of standard exponential rate equations, or are they non-standard examples?"

- The exponentials in the Boltzmann functions provided some numerical issues in our training runs. Upon further reflection, we agree that this is not necessarily a feature of the functions themselves, but some combination of addition of random effects, which may take extreme values, along with potentially large steps taken by the BFGS optimizer that we used. Therefore, this statement has been removed.

"Lines 37-38 coupled with 106-109. Citations should be utilized here. What does expensive mean? The authors claim that this new model formulation is simpler, more efficient, fast and more powerful. It is unclear what metrics are used for defining and indicating that the techniques used to generate HH equations are slow. It is also unclear if this is discussing the formulation of the model itself, or the fitting process or both."

- Even though we strongly believe our approach would be faster (in significant part due to fast ODE solving in Julia), the reviewer correctly notes that we do not show data to prove this, therefore we removed claims that it is faster.

"Line 123 "These channel types were chosen based on a preliminary inspection of the number of cell recordings that showed a good signal to noise ratio in a sufficient number of recordings over different temperatures " What does a good signal to noise ratio in a sufficient number of recordings mean? This should be made precise."

-Rephrased to clarify.

"Line 360 Authors should justify why i=2 gates was used. In the standard HH equations 4 gates are used due to the relationship with the protein composition and number of alpha protein subunits which undergo conformational changes to permit the passage of ions. N=4 was found to be used based on biophysical principles/mechanisms not arbitrarily. Here i=2 likely gives 1 activation and 1 inactivation gate type kinetics (which in fact is what the results end up showing), but it is unclear why 4 (to match Hodgkin Huxley and the protein subunit composition) or 1 (for simplicities sake) wasn't chosen instead. An interesting speculation would be how adding additional gates would impact the instances where m1 has a mix of inactivating and activating behaviour. Would this be broken up into two distinct gates?"

- We are not drawing and do not believe that it is reasonable to draw parallels between the number of gates and the protein structure, $i = 2$ was the simplest

model that fit the data, adding $i = 3$ did not provide significant improvements (data not shown). Moreover, we do not believe that Hodgkin and Huxley chose $i = 4$ because of the biophysical channel principles, but, quoting page 506 of their 1952 paper: "If g_k is used as a variable the end of the record can be fitted by a first-order equation but a third- or fourth-order equation is needed to describe the beginning. A useful simplification is achieved by supposing that g_k is proportional to the fourth power of a variable which obeys a first-order equation." and page 507 from the same paper (just below them defining g_k to have $i = 4$): "These equations may be given a physical basis if we assume that potassium ions can only cross the membrane when four similar particles occupy a certain region of the membrane." Reflecting on their work in 1976, Hodgkin wrote "As soon as we began to think about molecular mechanisms it became clear that the electrical data would by itself yield only very general information about the class of system likely to be involved. So we settled for the more pedestrian aim of finding a simple set of mathematical equations which might plausibly represent the movement of electrically charged gating particles." (*J. Phys.* 263:1–21). So it would appear that they chose $i = 4$ because it fit the data and then gave it a loose physical meaning, rather than choosing $i = 4$ because they knew that the potassium channel had four subunits.

Our speculation is that most likely adding more gates would break apart current gates with a mix of behaviours due to regularization, but this question is left for future research.

"Line 491 What do authors mean by "appropriate". Does this simply mean "result in the best fit?". What does constructed by hand mean? A citation would be helpful here to point out which types of models have biophysical support and which don't. Which types of models are constructed by hand and which are derived from first principles? Are these model types being grouped together or considered separately?"

- Added a clarification that "appropriate" simply means "resulting in the best fit". Unfortunately most model descriptions are not good enough to determine which models have significant biophysical support and which do not, as one may argue, with the benefit of hindsight, that the Hodgkin & Huxley model had biophysical support, whereas the authors may not have intended it and simply went with the best fits. All other things being equal, a model with biophysical support would be preferred over one without biophysical support, but – especially for the purposes of data-driven functional modelling – we feel a stronger constraint on model selection is goodness of fit.

"Lines 660-664, Again the authors should emphasize or recognize that good HH models (albeit not all modeling which is claimed to be in the style of HH are good) are based on biophysical properties where the exponents and number of gating particles is determined from experimental data and the protein level structure. This is why the more general Markov structure (which reduces to the HH formalism) is useful in helping readers see the channel structure. Voltage clamp data enables experimentalists to identify what types of gates should exist (activation, inactivation), and then analyzing protein structure with imaging techniques helps determine the number of particles. This is no more of an assumption than the authors choosing to use 2 gating particles."

- We can accept that many (if not most) HH models have rate coefficients constrained to some extent by biophysics, in particular transition state theory, (e.g. "Ion Chan-

nel Modeling beyond State of the Art: A Comparison with a System Theory-Based Model of the Shaker-Related Voltage-Gated Potassium Channel Kv1.1" (2022), Cells, 11(2), 239). However, we feel it is too strong to state that the number of all gating particles is *determined* from protein level structure

"Line 632. A functional form of Ti is given here in terms of reaction rates, but these rates are not described earlier in the text. While these rate functions alpha and beta are consistent with HH modeling, it isn't clear that the SciML framework is producing an alpha and beta term. Figure 10, the scale on the Time constant plots makes it difficult for the readers to parse out how big some of these time constants are and thus how important this activation variable actually is."

- Added a derivations involving alpha and beta functions in Equations 9-13. We recognize the limitations of Figure 10 and agree that in some cases the absolute sizes of time constants are difficult to parse out, but for some channels the time constants, depending on the temperature, can vary by three orders of magnitude. Therefore, choosing a scale that would provide enough detail for all of them is not feasible.

We again thank the Reviewers for their time (in particular the third reviewer for their thought provoking comments), insightful feedback and contributing to the scientific community. We hope that our clarifications and revisions address their feedback satisfactorily.

Many thanks for your consideration,

Domas Linkevicius
