# Supplementary material for: One model to rule them all: Unification of voltage-gated potassium channel models via deep non-linear mixed effects modelling
Source: PLoS Comput Biol. 2026 Apr 27;22(4):e1013078. doi: 10.1371/journal.pcbi.1013078 (PMC13143182; doi:10.1371/journal.pcbi.1013078)
Supplement: Attachment — Submitted filename: Kv_channel_models_PLoS_rebuttal_letter_2nd.pdf [file pcbi.1013078.s005.pdf]

School of informatics

THE UNIVERSITY OF EDINBURGH

10 March 2026

INSTITUTE *for* ADAPTIVE *and* NEURAL COMPUTATION

SCHOOL *of* INFORMATICS
The University of Edinburgh
The Informatics Forum
10 Crichton Street
Edinburgh EH8 9AB

Telephone 0131 650 3099
Fax 0131 650 6899
E-mail D.Linkevicius@sms.ed.ac.uk

Quentin Clairon, PhD
Guest Editor
PLOS Computational Biology

Hugues Berry
Section Editor
PLOS Computational Biology

Manuscript submission: "One model to rule them all: unification of voltage-gated potassium channel models via deep non-linear mixed effects modelling"
Domas Linkevicius, Angus Chadwick, Melanie I. Stefan & David C. Sterratt

Dear Dr Clairon and Prof Berry,

We are thankful to the reviewers for their effort, address the new set of points raised and clarify our previously unsatisfactory rebuttals.

Reviewer #3 wrote:

> Line 812 'it is a subclass of the broader Markov models, where the gating particles may not be independent.' This should be clarified, Markov models are inherently independent.

We have changed the wording to "... it is a subclass of the broader Markov models, where the interaction of gating particles in determining the open channel fraction may be more complex than a simple product." to clarify that we did not mean breaking of the Markov property.

Reviewer #1 wrote:

> Authors have provided a sequence of comments that follow my remarks stream. However, I request to include in that list the modifications done in the text. Besides, mark in some color the changes done in the main document. It is quite annoying to go back and forth trying to locate the changes (if any).

We provide these details below, loosely grouping points by type of response: specification of changes within the text, points we feel do not require modifications of the text, and points we found to be slightly vague and interpretable in multiple ways.

Modifications to the text:

> 0. After the Introduction and up to the Methods section, the authors only mention what they do, but they don't explain why or from what references they draw their writing. This is particularly true of the parameters chosen, the methods used, and the general sequence of the process and the document. Some explanations are vague. It

would be ok if some additional figures could be included to be clearer to the readers.

We added the following paragraph to the introduction (Note that citation numbers relate to the reference list at the foot of this letter):

"The feature of the mixed effect frameworks to include flexible functions lends itself to integration with machine learning approaches. One of the first applications of mixed effects along with neural networks used a ResNet architecture, with the resulting approach called a mixed effects network (MeNet)[1]. Using a MeNet authors processed gaze estimation image data and then used the extracted features within a linear mixed effects framework. Since the gaze estimation data they used were obtained by repeated measurements from the same participants, taking into account this dependency between the data resulted in up to 20% improvements in errors over the state of the art. A generalized version of the MeNet approach is neural mixed effects (NME) [2]. In NME, instead of placing a neural network as a feature extractor (a fixed effect), there are random effects on the neural network weights, using both person-generic parameters $\bar{\theta}$ and person-specific parameters θ_n, using $\bar{\theta} + \theta_n$ as the actual neural network weights. NMEs outperformed MeNets on six different tasks. Therefore, the NME approach, individualization of the neural network weights, provided additional benefits to performance. Finally, the DeepNLME approach [3], instead of putting a random effect on each parameter of the neural net, uses random effects as inputs to the neural network, in addition to its regular inputs. Thus the neural net weights are fixed effects, yet the resulting functions are individualized for the nth observed entity. Approaches that integrate other machine learning models, such as random forests, can also be used combination with mixed effects [4, 5, 6, 7]."

1. Please indicate the main characteristics of the data, to prevent the reader from obtaining the reference.
2. Minimum, maximum, average of cell recordings?

We have added the following table:

	Initial number of cells				Number of cells after preprocessing			
Channel type	Total	15°C	25°C	35°C	Total	15°C	25°C	35°C
All types	3171	807	1614	750	662	171	330	161
$K_v1.1$	298	68	163	67	55	14	27	14
$K_v1.2$	210	57	110	43	45	12	23	10
$K_v1.3$	363	73	203	87	74	15	41	18
$K_v1.4$	229	57	120	52	47	12	24	11
$K_v1.5$	249	37	179	33	52	8	36	8
$K_v1.6$	134	35	62	37	28	7	13	8
$K_v1.8$	91	24	49	18	20	6	11	3
$K_v2.1$	102	32	56	14	23	7	12	4
$K_v2.2$	115	20	63	32	25	4	14	7
$K_v3.1$	120	42	45	33	26	9	9	8
$K_v3.2$	153	34	90	29	33	8	18	7
$K_v3.3$	233	36	123	74	49	8	26	15
$K_v3.4$	154	41	72	41	33	9	15	9
$K_v4.1$	102	31	49	22	21	6	11	4
$K_v4.2$	96	32	33	31	19	7	7	5
$K_v4.3$	120	39	63	18	24	8	13	3
$K_v10.1$	113	38	49	26	25	8	11	6
$K_v10.2$	61	19	13	29	15	4	4	7
$K_v12.1$	159	60	50	49	34	12	11	11
$K_v12.3$	69	32	22	15	14	7	4	3

> 3. WHAT is the 'sufficient' number of recordings ('sufficient' with respect to what and why?)
> 4. From (3): Why at those specific temperatures?

Changed from: "These channel types were chosen based on a preliminary inspection of the number of cell recordings that showed a good signal to noise ratio in a sufficient number of recordings over different temperatures (15°C, 25°C and 35°C)"

to:

"These channel types were chosen based on a preliminary visual inspection of the quality of recordings (e.g. absence of systematic artefacts) and the existence of multiple recordings at each of the different temperatures (15°C, 25°C and 35°C, the only temperatures used in Ranjan et al. [8])."

> 6. Part: Data Processing. Authors claim that: 'In order to make the data in Ranjan et al. [4] suitable for model training we undertook 166 a series of data processing steps (Figure 1). First, we filtered out inconsistent data. We 167 then applied a time series smoothing algorithm to reduce the noise levels, followed by 168 setting the current baseline to 0, normalization of the current, rescaling, exclusion of 169 systematic data artifacts and downsampling'.
> Again, why those numbers and WHAT IS THE SMOOTHING algorithm? Why those parameters?

We clarified that more details about each data processing step are given in the sections that

describe them and that the parameters were chosen such that the data would be visually as similar to the originally used data, except for immediately identifiable artefacts. Also, the original text included "It implements a smoothing function called `denoise` which transforms the signal using the discrete cosine transform and convolves it with an Gaussian function (similar to the method outlined in [9])."

7. WHY this...? All data processing, modelling and 170 analyses in this paper were performed using the Julia v1.10.4 programming 171 language.

We added "All data processing, modelling and analyses in this paper were performed using the `Julia v1.10.4` programming language [10] due to the superior ordinary differential equation solver speeds of `DifferentialEquations.jl` compared to packages available in other programming languages, like Python or MATLAB [11, 12], as well as the existence of a performant nonlinear mixed effects package with machine learning capabilities – `Pumas.jl` [13] and `DeepPumas.jl` [14]."

8. Why to use MAPE and not other measure or more than 1 measure? Why 1 measure is ok and why only one parameter is ok? How can you justify this?

We added "We compared the sweeps from other protocols to the relevant parts of the activation protocol using the mean absolute percentage error (MAPE), which is a well-known comparison measure in time series forecasting [15, 16]."

9. Authors say that: "The dynamical parameters pn are then fed into the structural model (e.g. an ordinary 301 differential equation (ODE) system)". But where is the ODE system?

The ODE system is given in Equation (3) in the original text, just below the text referenced by the reviewer. We clarified that u' denotes the time derivative.

13. Line 308: What do you mean with: 'the appropriate variables'?

We changed "the appropriate variables are derived and passed through a Gaussian observational model to account for observational noise" to "the observable quantities are derived from the values obtained numerically and passed through a Gaussian observational model to account for observational noise"

19. The optimization problem should formally be posed.

The optimization problem is formally posed in equations 5 through 7 (as it is multi-stage optimization).

21. How to be sure that the NN algorithm actually converges?

As shown in S1 appendix, since the training loss does not decrease with further training iterations, this means that it has converged locally.

Points we feel do not require modifications of the manuscript:

10. From (9), why not to use PDEs instead of ODEs? A good discretization could

yield good results.

Usage of PDEs in this context, especially in compartmental modelling of neurons, is outside of the scope of the present work. In the current work the cells conducting current have regular shapes and therefore can be approximated as points, so usage of PDEs is not necessary.

11. From (9,10), how do you know that the set of ODE actually have a solution?

The reviewer correctly notes that the ODEs do not have an analytical solution, but that is not a significant problem as usage of numerical solvers in such modelling applications is standard practice.

12. Depending of the parameters of the ODE, the ODE may reflect some ill-posed issue, particularly with small values of the coefficients.

It is true that the resulting ODE system may be stiff, but this is addressed by using an auto-switching ODE solver that uses a stiff solver when the parameters result in a stiff system, and a non-stiff solver when the parameters produce a non-stiff system.

15. How do ensure that your data has a joint Gaussian function?

We do not require that the data has a joint Gaussian function, as the Gaussian observational model is per-point, rather than joint, as indicated by the subscript n in Eq. 4. Also, Figure 8 provides favourable diagnostics that the error model we use is appropriate.

16. About eq. (4), it has been proven that the uncertainty directly from Gaussian Processes Regression is irrelevant to the cohort heterogeneity in general. What happens in your case about this?

The only source we can find for this finding is in Xu et al (2021, "The pitfalls of using Gaussian Process Regression for normative modeling", PLoS ONE) who state "we show that the uncertainty directly from Gaussian Processes Regression is irrelevant to the cohort heterogeneity in general." However, the "cohort heterogeneity" modelled in that paper is different from the problem manifest in the data we are analysing. It is not clear to us how Gaussian Process Regression relates to to universal differential equations in an NLME context, as considered in our work.

17. In real life, returns are not symmetrically distributed and exhibit extremes not captured well by the bell-shaped curve of the Gaussian model.

We are not sure what the "returns" referred to here are, though this statement does appear to apply to financial scenarios, see, e.g., this article about the use of the Gaussian in finance, which states "In real life, returns are not symmetrically distributed and exhibit extremes not captured well by the bell-shaped curve of the Gaussian model." (`https://campus.datacamp.com/courses/financial-analytics-in-google-sheets/monitoring-the-distribution-of-returns?ex=14`). That said, it is true that in NLME applications a proportional, rather than additive error model is used, accounting better for extreme values. However, it is necessary only when the extreme values are orders of magnitude apart, which was not the case in the current data set.

18. Line 314: Maximum likelihood estimates can be heavily biased for small samples,

> thus the optimality properties may not apply for small samples. Maximum likelihood can be sensitive to the choice of starting value. What happens with yours?

We believe that the small sample regime does not apply to our case, since we have a large amount of data compared to general NLME applications. Also, our work is concerned with applying the NLME methodology to model the Kv gating; for the analysis usage of ML estimates under various data regimes we would refer the reader to the source publication for `Pumas.jl`.

> 20. How do you guarantee, in advance, that such optimum value will be found? (global, local...).

Guaranteeing that in advance for neural network based modelling is generally not possible, but requires exploration of hyper-parameters, which we show in S1 appendix. Also, [17] show that individual gating particle equilibrium functions are structurally non-identifiable. Therefore, we can say with confidence that any minima are local, rather than global.

> 22. What is the computational and algorithmic complexity of the whole algorithm?

In this work we apply the NLME methodology. For features of the algorithm, such as its complexity, we refer the reader to the `Pumas.jl` source publication.

> 24. From (16-18): Unlike ML estimators, the MAP estimate is not invariant under reparametrization. Switching from one parametrization to another involves introducing a Jacobian that impacts on the location of the maximum. In contrast, Bayesian posterior expectations are invariant under reparametrization. Please comment about this."

In this work we do not endeavour to estimate parameters, as neural network parameters are not inherently physically meaningful. Therefore, while the reviewer raises a valid and interesting point, it is not immediately relevant to the current work.

Points that we found to lack clarity:

> 5. Authors provide and describe parameters with no rationale nor references. WHY do you work exactly with those values?

We are not clear which parameters the reviewer is referring to, but we would be happy to address this point if more details are specified.

> 14. Eq. 4 implies a lot of things but you do not explain anything else.

We believe that we have addressed all relevant implications from this equation in the text, and the reviewer has given no specific indications of what, in their opinion, is missing.

> 23. Why not to conduct a statistical test to test the classification results?

Could the reviewer be more specific on which results warrant statistical tests pertinent to classification, as we do not believe we have conducted any classification in this work.

We again thank the Reviewers for their time, we hope that these additional clarifications address

their feedback satisfactorily.

Many thanks for your consideration,

Domas Linkevicius

References

[1] Yunyang Xiong, Hyunwoo J. Kim, and Vikas Singh. Mixed effects neural networks (menets) with applications to gaze estimation. In *2019 IEEE/CVF Conference on Computer Vision and Pattern Recognition (CVPR)*, pages 7735–7744, 2019. doi: 10.1109/CVPR.2019.00793.

[2] Torsten Wörtwein, Nicholas B. Allen, Lisa B. Sheeber, Randy P. Auerbach, Jeffrey F. Cohn, and Louis-Philippe Morency. Neural mixed effects for nonlinear personalized predictions. In *Proceedings of the 25th International Conference on Multimodal Interaction*, ICMI '23, page 445–454, New York, NY, USA, 2023. Association for Computing Machinery. ISBN 9798400700552. doi: 10.1145/3577190.3614115. URL `https://doi.org/10.1145/3577190.3614115`.

[3] Carl Julius Martensen, Niklas Korsbo, Vijay Ivaturi, and Sebastian Sager. Data-driven discovery of feedback mechanisms in acute myeloid leukaemia: Alternatives to classical models using deep nonlinear mixed effect modeling and symbolic regression. *bioRxiv*, 2024. doi: 10.1101/2024.06.17.599366. URL `https://www.biorxiv.org/content/early/2024/06/19/2024.06.17.599366`.

[4] Ahlem Hajjem, François Bellavance, and Denis Larocque. Mixed effects regression trees for clustered data. *Statistics & Probability Letters*, 81(4):451–459, 2011. ISSN 0167-7152. doi: https://doi.org/10.1016/j.spl.2010.12.003. URL `https://www.sciencedirect.com/science/article/pii/S0167715210003433`.

[5] Ahlem Hajjem, François Bellavance, and Denis Larocque. Mixed-effects random forest for clustered data. *Journal of Statistical Computation and Simulation*, 84(6):1313–1328, 2014. doi: 10.1080/00949655.2012.741599. URL `https://doi.org/10.1080/00949655.2012.741599`.

[6] Ahlem Hajjem, Denis Larocque, and François Bellavance. Generalized mixed effects regression trees. *Statistics & Probability Letters*, 126:114–118, 2017. ISSN 0167-7152. doi: https://doi.org/10.1016/j.spl.2017.02.033. URL `https://www.sciencedirect.com/science/article/pii/S0167715217300895`.

[7] Shuwen Hu, You Gan Wang, Christopher Drovandi, and Taoyun Cao. Predictions of machine learning with mixed-effects in analyzing longitudinal data under model misspecification. *Statistical Methods and Applications*, 32:681–711, 2023. ISSN 1613981X. doi: 10.1007/s10260-022-00658-x.

[8] Rajnish Ranjan, Emmanuelle Logette, Michela Marani, Mirjia Herzog, Valérie Tâche, Enrico Scantamburlo, Valérie Buchillier, and Henry Markram. A kinetic map of the homomeric voltage-gated potassium channel (kv) family. *Frontiers in Cellular Neuroscience*, 13, 8 2019. ISSN 16625102. doi:

10.3389/fncel.2019.00358.

[9] Abraham. Savitzky and M. J. E. Golay. Smoothing and differentiation of data by simplified least squares procedures. *Analytical Chemistry*, 36(8):1627–1639, 1964. doi: 10.1021/ac60214a047. URL https://doi.org/10.1021/ac60214a047.

[10] Jeff Bezanson, Alan Edelman, Stefan Karpinski, and Viral B Shah. Julia: A fresh approach to numerical computing. *SIAM Review*, 59(1):65–98, 2017. doi: 10.1137/141000671. URL https://epubs.siam.org/doi/10.1137/141000671.

[11] Christopher Rackauckas and Qing Nie. Confederated modular differential equation apis for accelerated algorithm development and benchmarking. *Advances in Engineering Software*, 132:1–6, 2019.

[12] Christopher Rackauckas and Qing Nie. Differentialequations.jl – a performant and feature-rich ecosystem for solving differential equations in julia. *The Journal of Open Research Software*, 5(1), 2017. doi: 10.5334/jors.151. URL https://app.dimensions.ai/details/publication/pub.1085583166andhttp://openresearchsoftware.metajnl.com/articles/10.5334/jors.151/galley/245/download/. Exported from https://app.dimensions.ai on 2019/05/05.

[13] Chris Rackauckas, Yingbo Ma, Andreas Noack, Vaibhav Dixit, Patrick Kofod Mogensen, Chris Elrod, Mohammad Tarek, Simon Byrne, Shubham Maddhashiya, José Bayoán Santiago Calderón, Michael Hatherly, Joakim Nyberg, Jogarao V.S. Gobburu, and Vijay Ivaturi. Accelerated predictive healthcare analytics with pumas, a high performance pharmaceutical modeling and simulation platform. *bioRxiv*, 2022. doi: 10.1101/2020.11.28.402297. URL https://www.biorxiv.org/content/early/2022/03/20/2020.11.28.402297.

[14] Ivaturi Vijay Rackauskas Christopher. Neural-embedded nonlinear mixed effects models (nenlme) in pumas.jl. In *American Conference on Pharmacometrics*, 2019.

[15] Spyros Makridakis and Michèle Hibon. The M3-Competition: Results, conclusions and implications. *International Journal of Forecasting*, 16(4):451–476, 2000. ISSN 01692070. doi: 10.1016/S0169-2070(00)00057-1. URL https://linkinghub.elsevier.com/retrieve/pii/S0169207000000571.

[16] Rob J. Hyndman and Anne B. Koehler. Another look at measures of forecast accuracy. *International Journal of Forecasting*, 22(4):679–688, October 2006. ISSN 01692070. doi: 10.1016/j.ijforecast.2006.03.001.

[17] Olivia J. Walch and Marisa C. Eisenberg. Parameter identifiability and identifiable combinations in generalized hodgkin–huxley models. *Neurocomputing*,

199:137–143, 2016. ISSN 0925-2312. doi: https://doi.org/10.1016/j.neucom.2016.03.027. URL https://www.sciencedirect.com/science/article/pii/S0925231216300479.
